# High-sensitivity cardiac troponin and the early rule out of myocardial infarction: time for action

**DOI:** 10.1136/heartjnl-2020-316811

**Published:** 2020-04-27

**Authors:** Andrew R Chapman, Nicholas L Mills

**Affiliations:** BHF Centre for Cardiovascular Science, The University of Edinburgh, Edinburgh, UK

**Keywords:** acute coronary syndromes, acute myocardial infarction, meta-analysis

Chest pain is one of the most frequent presenting complaints in patients attending the emergency department, with almost 1 million attendances across the UK every year.^1^ The priority of the attending clinician is to promptly reach a diagnosis, as early intervention with medical therapy has been shown to reduce morbidity and mortality in patients with myocardial infarction. However, as few as 1 in 10 patients with chest pain actually have a diagnosis of myocardial infarction, with the majority presenting due to alternative diagnoses. Therefore, the safe and early rule out of myocardial infarction may be helpful by facilitating a refocused clinical evaluation, with targeted investigations for alternative differential diagnoses. This can also provide confidence that patients with typical anginal symptoms have not had myocardial infarction and may be safely triaged for outpatient investigation.

High-sensitivity cardiac troponin assays were first introduced across Europe and Australasia in 2010. There are now several high-sensitivity assays available for use, all of which offer a magnitude improvement in performance over the previous generation. A number of diagnostic algorithms have been written which exploit the performance of these assays for detection of cardiac troponin at low concentration. For example, in patients with suspected acute coronary syndrome with a non-ischaemic ECG and a high-sensitivity cardiac troponin I or T concentration <5 ng/L (measured using the Abbott ARCHITECT or Siemens Atelleca hs-cTnI, or the Roche Elecsys hs-cTnT assays), both negative predictive value and sensitivity are extremely high, missing as few as 1 in every 200 patients tested and stratifying between 30% and 50% of patients as low risk.^2–4^ The High-Sensitivity Cardiac Troponin on Presentation to Rule-Out Myocardial Infarction (HiSTORIC) trial evaluated this approach in a stepped-wedge cluster randomised controlled trial, reporting at the European Society of Cardiology (ESC) Congress in 2019, demonstrating implementation of an early rule-out pathway for myocardial infarction reduced length of stay and hospital admission without increasing adverse cardiac events.

The ESC 0/1 hour algorithm is one approach to the risk stratification of patients with suspected acute coronary syndrome. In this pathway, patients without ischaemia on the ECG are stratified based on their presentation and 1 hour troponin concentrations using assay-specific thresholds. In patients with very low troponin concentrations below the ‘rule-out’ threshold with at least 3 hours of symptoms, myocardial infarction is immediately ruled out on a single test. In those with troponin concentrations above a predefined ‘rule-in’ threshold, the likelihood of myocardial infarction is high, and patients are admitted for investigation and treatment. In all others, repeat troponin testing is performed at 1 hour after the initial sample to detect small but important rises in cardiac troponin concentration. If on serial testing the rule-in or rule-out thresholds are not reached, patients are observed for myocardial ischaemia and should undergo peak troponin testing to guide decision making.

Chiang *et al* 5 present findings from a systematic review and meta-analysis of studies evaluating the performance of the ESC 0/1 hour algorithm in patients with suspected acute coronary syndrome. They included 11 014 patients from 15 studies and 10 individual cohorts across Europe, the USA, Japan, Thailand and China, employing bivariate random-effects methodology for a primary outcome of index myocardial infarction and secondary outcome of major adverse cardiac event or death from any cause.

**Figure 1:**
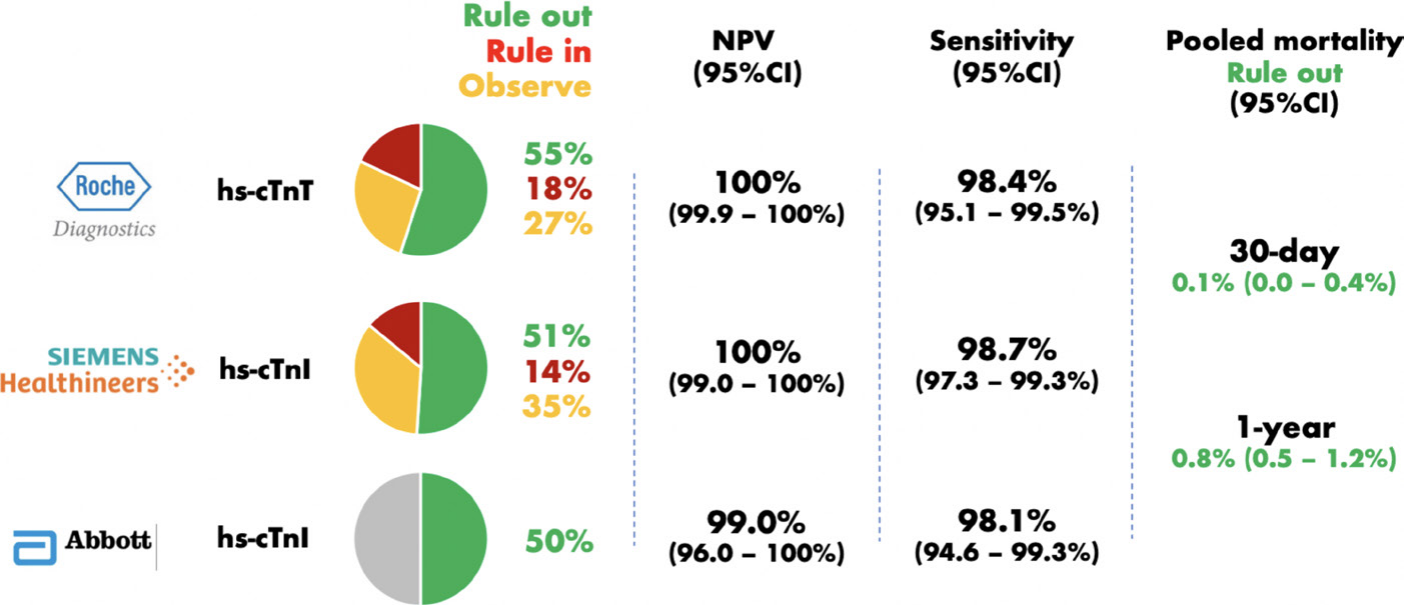
Comparison of the performance of the European Society of Cardiology (ESC) Congress 0/1 hour algorithm for the rule out of index myocardial infarction across three high-sensitivity cardiac troponin assays. Note no meta-estimates were obtained for either rule-in or observational zone performance using the Abbott hs-cTnl assay due to insufficient study numbers. NPV, negative predictive value.

The authors found similar diagnostic performance across three commercially available high-sensitivity cardiac troponin assays for the exclusion of index myocardial infarction. The Roche hs-cTnT assay identified 55% of patients as low risk, with a pooled sensitivity of 98.4% (95% CI 95.1% to 99.5%) and negative predictive value (NPV) of 100% (95% CI 99.9% to 100%). The Abbott hs-cTnI assay identified 50% as low risk, with a sensitivity and NPV of 98.1% (95% CI 94.6% to 99.3%) and 99% (95% CI 96.0% to 100%), and the Siemens hs-cTnI assay identified 51% of patients as low risk, with a sensitivity and NPV of 98.7% (95% CI 97.3% to 99.3%) and 100% (95% CI 99% to 100%). In patients triaged to rule out, the mortality at 30 days and at 1 year was low, at 0.1% (95% CI 0.0% to 0.4%) and 0.8% (95% CI 0.5% to 1.2%), respectively (figure 1).

The rule-in performance of the ESC 0/1 hour algorithm was also evaluated. The Roche hs-cTnT assay ruled in 18% of patients with a positive predictive value (PPV) of 51% (95% CI 31% to 71%), whereas the Siemens hs-cTnI assay ruled in 14% of patients with a PPV of 73% (95% CI 69% to 77%). As only two studies evaluated the performance of the Abbott hs-cTnI for rule in, no meta-estimates were obtained.

Notably, the proportion of patients triaged towards the observation zone differed markedly across cohorts, from 22.4% (TRAPID Cohort; Roche hs-cTnT) to 37.1% (HIGH-US Cohort; Siemens hs-cTnI). Patients in this category were at high risk of adverse outcomes. In a secondary analysis evaluating the future risk of MACE or death, at 30 days, the pooled event rate for patients in the observation zone was equivalent to the event rate in the rule-out population at 1 year (event rate 0.7%, 95% CI 0.3% to 1.2% in the observation group at 30 days vs event rate 0.8%, 95% CI 0.6% to 1.0%). Furthermore, at 1 year, the event rate in patients within the observation zone was equivalent to those who were ruled in with a high probability of myocardial infarction (event rate 8.1%, 95% CI 6.1% to 10.4% in the observation group vs 10%, 95% CI 7.8% to 12.4% in the rule-in group).

This analysis provides important insights into the performance of the ESC 0/1 hour algorithm across different healthcare settings. There are several strengths. The authors conducted a systematic review and included only prospective cohort studies in patients presenting to the emergency department with suspected acute coronary syndrome, where high-sensitivity cardiac troponin concentrations were measured using a commercially available assay, and the diagnosis of myocardial infarction was adjudicated according to the Universal Definition. The prevalence of myocardial infarction varied from 2% (Parkland, USA) to 32% (Beijing, China). Where multiple publications emerged from the same cohorts, the authors included the study with the largest sample size or most complete dataset.

As with all meta-analyses there are some limitations. First, studies did not use identical criteria for recruitment, with some cohorts excluding patients with missing 1 hour samples, or those with comorbidity such as end-stage renal failure. Second, some cohorts evaluated the ESC 0/1 hour algorithm against an outcome including a diagnosis of type 2 myocardial infarction. Type 2 myocardial infarction occurs in the context of another acute illness where there is myocardial oxygen supply or demand imbalance. These patients are by definition unwell and require hospitalisation for assessment and treatment, and in clinical practice are not considered for discharge. Pathways such as the ESC algorithm were not intended for this purpose. Third, the authors did not have access to individual patient-level data, and while the ESC guidelines state this algorithm should only be applied in patients without myocardial ischaemia on the ECG, all cohorts did not necessarily apply these criteria, and this may have adversely affected performance. Fourth, the authors included patients within the original ESC 0/1 hour algorithm derivation cohorts in some analyses. They attempted to mitigate for this by conducting a sensitivity analysis with these cohorts excluded, and diagnostic performance was similar to overall estimates. Finally, all patients included in this meta-analysis were recruited as part of observational cohort studies, where management was not guided by the ESC 0/1 hour algorithm, and individual management decisions may have influenced patient outcomes.

Overall, the authors conclude that the ESC 0/1 hour algorithm may not have the required diagnostic performance if a sensitivity of 99% is mandated, but is this truly required in practice? Very few tests in clinical practice have achieved such a goal. There are strengths and limitations of using either sensitivity or NPV as the primary safety metric, but in the majority of cohorts, the ESC 0/1 hour algorithim performed very well. Study-specific factors may well have influenced the performance observed in the two cohorts which were outliers (Barcelona cohort; sensitivity 88%, 95% CI 68.8% to 97.5%, NPV 96.9%, 95% CI 91.2% to 99.4%; Beijing cohort; sensitivity 92.3%, 95% CI 84.8% to 96.9%, NPV 91.3%, 95% CI 82.8% to 96.4%).

Notably, the ESC 0/1 hour algorithm has already been evaluated in a prospective randomised controlled trial.^6^ Chew *et al* randomly assigned 3378 patients with suspected acute coronary syndrome to either the ESC 0/1 hour hs-cTnT pathway, or a standard care arm using hs-cTnT at the 99th centile. They demonstrated an increased rate of discharge from the emergency department with the 0/1 hour algorithm (45.1% vs 32.3%, p<0.001) and a reduced length of stay (4.6, IQR (3.4–6.4) hours vs 5.6 (4.0–7.1) hours, p<0.001). The ESC 0/1 hour pathway had an overall NPV of 99.6% (99.0%–99.9%) for 30-day death or myocardial infarction, and was non-inferior to standard care. Similar findings were observed in a non-randomised, prospective international multicentre implementation study by Twerenbold *et al*.^7^ In 2296 patients with suspected acute coronary syndrome, the ESC 0/1 hour algorithm was followed in 94% of cases, with a median length of stay 2.5 (2.3–4.0) hours. The majority of patients (62%) were ruled out in the emergency department, with just 0.2% (3/1420) of patients experiencing a major adverse cardiovascular event at 30 days.

There are a number of well-validated approaches which apply cardiac troponin as a continuous variable, all of which offer a magnitude improvement in safety over those using troponin as a binary variable at the 99th centile.^8–10^ There are strengths and limitations of each, but the choice of algorithm is best determined at a local level in conjunction with both the clinical and laboratory service. The decision to admit or discharge will always be taken by a physician using all available clinical information, including the ECG, results from other investigations and the presence of ongoing symptoms. While pathways do simplify decision making, they do not replace clinical judgement, and a final diagnosis should always be sought.

It is difficult to overlook the extreme pressures that healthcare systems worldwide are currently experiencing as a result of the Coronavirus pandemic. While many emergency departments will already have implemented rapid rule-out pathways for acute myocardial infarction, some have not, particularly those in the USA. There is a plethora of data evaluating high-sensitivity troponin testing in patients with suspected acute coronary syndrome, including two prospective, randomised controlled trials, demonstrating very low rates of future myocardial infarction or death as far as 1 year after index presentation. It is time to take advantage of these strategies to reduce overcrowding, mitigate the associated risks of hospitalisation and prevent harm.
